# Anti-Inflammatory Action of Dietary Wild Olive (Acebuche) Oil in the Retina of Hypertensive Mice

**DOI:** 10.3390/foods10091993

**Published:** 2021-08-25

**Authors:** Álvaro Santana-Garrido, Claudia Reyes-Goya, Santiago Milla-Navarro, Pedro de la Villa, Helder André, Carmen M. Vázquez, Alfonso Mate

**Affiliations:** 1 Departamento de Fisiología, Facultad de Farmacia, Universidad de Sevilla, 41012 Sevilla, Spain; asgarrido@us.es (Á.S.-G.); crgoya@us.es (C.R.-G.); vazquez@us.es (C.M.V.); 2 Epidemiología Clínica y Riesgo Cardiovascular, Instituto de Biomedicina de Sevilla (IBIS), Hospital Universitario Virgen del Rocío/Consejo Superior de Investigaciones Científicas/Universidad de Sevilla, 41013 Sevilla, Spain; 3 Department of Systems Biology, University of Alcalá, 28871 Madrid, Spain; santiago.milla@edu.uah.es (S.M.-N.); pedro.villa@uah.es (P.d.l.V.); 4 Instituto Ramón y Cajal de Investigación Sanitaria (IRYCIS), 28034 Madrid, Spain; 5 Department of Clinical Neuroscience, St. Erik Eye Hospital, Karolinska Institutet, 11282 Stockholm, Sweden; helder.andre@ki.se

**Keywords:** acebuche, arterial hypertension, inflammation, olive oil, retina, wild olive tree

## Abstract

Inflammation plays a crucial role in the course of eye diseases, including many vascular retinopathies. Although olive oil is known to have beneficial effects against inflammatory processes, there is no information available on the anti-inflammatory potential of the wild olive tree (namely, acebuche (ACE) for the primitive Spanish lineages). Here we investigate the anti-inflammatory effects of ACE oil in the retina of a mouse model of arterial hypertension, which was experimentally induced by administration of L-NAME (NG-nitro-L-arginine-methyl-ester). The animals were fed supplements of ACE oil or extra virgin olive oil (EVOO, for comparative purposes). Retinal function was assessed by electroretinography (ERG), and different inflammation-related parameters were measured in the retina and choroid. Besides significant prevention of retinal dysfunction shown in ERG recordings, ACE oil-enriched diet upregulated the expression of the anti-inflammatory markers PPARγ, PPARα and IL-10, while reducing that of major proinflammatory biomarkers, IL-1β, IL-6, TNF-α and COX-2. This is the first report to highlight the anti-inflammatory properties of an ACE oil-enriched diet against hypertension-related retinal damage. Noteworthy, dietary supplementation with ACE oil yielded better results compared to a reference EVOO.

## 1. Introduction

Numerous authors have recognized the pivotal role of extra virgin olive oil (EVOO) (*Olea europaea* L.) in the context of the well-known Mediterranean diet, and the potential benefits of its multiple bioactive compounds [1,2]. In this sense, EVOO has been identified as a key to reducing the risk of various diseases [3,4,5]. The beneficial health outcomes derived from the regular consumption of olive oil rely on its nutritional components to which antioxidant, anti-inflammatory and antitumoral properties are ascribed.

The major constituents of EVOO include acyclglycerols, free fatty acids, pigments and phosphatides, among others. A high proportion of monounsaturated fats also defines its distinctive biochemical profile [6], of which oleic acid (C18:1) is probably the most studied [7]. Polyunsaturated fatty acids (PUFAs) include linoleic (C18:2) and α-linolenic (C18:3) acids, whereas saturated fatty acids (SFA) account only for 8–14% [8]. Interestingly, EVOO also contains a wide range of minor components including sterols, tocopherols, triterpenic and phenolic compounds, which are involved in a profusion of pathways involved in homeostasis, inflammation and redox state [9,10,11,12]. Consequently, regular consumption of EVOO has been proposed as a powerful nutraceutical tool to avert and mitigate cancer and cardiovascular or degenerative diseases [13,14], where noticeable contributions of hydroxytyrosol and its derivates (tyrosol, oleuropein and oleocanthal) have been reported [15]. Nonetheless, some authors have claimed the need to discover additional minor bioactive components of EVOO that might help understand its beneficial properties.

Unlike the renowned reports concerning the consumption of fruits and oil obtained from the common olive tree (*Olea europaea* var. *europaea*) in the setting of the Mediterranean diet, the information available on the wild olive tree (*Olea europaea* var. *sylvestris*)—namely, acebuche (ACE) for the primitive Spanish lineages—is very limited. Thus, although different studies have addressed the composition and therapeutic effects of EVOO, very little is known specifically about ACE oil, a product of growing interest in specific regions such as Andalusia, Spain. The conservation of genuine wild olive lineages is desirable from an environmental point of view; however, these olive varieties have remained largely under-exploited, because the fruit of the wild olive yields little oil compared to cultivated olives. Therefore, the small amounts of commercially available ACE oil are typically consumed as “gourmet” products that are generally recognized as spicier, more bitter and fruitier than standard EVOOs, possibly due to slight differences in minor compounds [16,17]. Some of the first evidences have attributed a higher content of tocopherols (e.g., vitamin E), sterols and triterpene acids in ACE oil than in EVOO, and also a higher amount of secoiridoid compounds relative to ortodiphenols in the former [18,19]. Moreover, ACE oil has been reported to have lower antigenic and allergenic capacities compared to cultivated olive trees [20].

Despite the importance of EVOO in protecting against cardiovascular diseases (CV), its potential role as a bioactive supplement able to counteract the development/progression of ocular diseases is not well established yet. Previous population studies have suggested that EVOO might be useful to delay the occurrence of age-related macular degeneration (AMD) [21,22,23], while in vivo and in vitro experiments described neuronal protection of EVOO components (e.g., hydroxytyrosol and oleuropein) in diabetic retinopathy (DR) [24,25,26,27]. In this regard, we have recently demonstrated a retinoprotective action of EVOO- and ACE oil-enriched diets, showing better results in the latter case, in a context of arterial hypertension (AH). The beneficial actions of these oils were ascribed to their capacity to counteract the progression of hypertensive eye disease by reducing superoxide anions (O_2_^·−^) and by modulating the enzymes NADPH oxidase and nitric oxide synthase, among others [19]. Therefore, ACE oil and EVOO-based diets could represent a strategical tool to reduce AH-related ocular damage in pathologies such AMD, DR or hypertensive retinopathy, among others. However, knowing the precise mechanisms responsible for the beneficial properties of olive oil consumption at the ocular level requires further research.

Along with oxidative stress, inflammation has been extensively studied as a key mechanism in the pathogenesis of hypertension, especially in the context of persistent AH [28,29]. The expression of major inflammatory biomarkers, including tumor necrosis factor-alpha (TNF-α) [30], cyclooxygenase-2 (COX-2) [31] and interleukin (IL) isoforms [32], is known to be modulated during the course of AH. In addition, it is well known that low-grade inflammation in AH is associated with several pathways involved in the development and progression of different ocular pathologies [33,34], including DR [35], glaucoma [36] and AMD [37], among others. While it is also noteworthy that the literature on the interplay between inflammation and AH supports the involvement of the former in hypertensive retinopathy [38] and DR [39], how hypertension contributes to inflammation and its relevance in the development/progression of major retinopathies has not been previously studied. Some of the components of EVOO mentioned above have been postulated as possible contributors to reduce the inflammation process in different diseases, including hydroxytyrosol [40], oleocanthal [41] or triterpenes such as ursolic and oleanolic acids [42], among others. Novel dietary and/or therapeutic strategies could help reduce retinal inflammation (whether or not related to AH) and describe the possible implication of AH in this regard, thus contributing to unveil hypertension-associated target organ damage.

The purpose of the current study was to test the ability of an ACE oil-enriched diet to counteract retinal inflammation, based on a plausible anti-inflammatory effect, in a rodent model of AH triggered by chronic administration of L-NAME (NG-nitro-L-arginine-methyl-ester). Visual function was estimated by electroretinography in unconscious hypertensive mice after a 6-week period of ACE oil administration. Moreover, the expression of peroxisome proliferator-activated receptors (PPARs) and that of inflammation-related biomarkers (namely, interleukin isoforms (IL-1β, IL-6 and IL-10), TNF-α and COX-2) was assayed by immunohistofluorescence in retinal/choroid layers, and by Western blotting and real-time PCR in retinal homogenates. Additional experiments were carried out in parallel substituting EVOO for ACE oil, for comparative analysis.

## 2. Materials and Methods

### 2.1. Study Design

The present study complies with the European Union (EU) Directive 2010/63/EU and the National (RD 53/2013) guidelines for the care and use of Laboratory animals and was approved by the relevant Institutional Animal Care and Use Committee (Dirección General de Producción Agrícola y Ganadería, Junta de Andalucía, reference #13/03/2019/031). 10–12-week-old male C57B/6J mice were supplied by the Center for Animal Production and Experimentation (University of Seville, Spain). The animals were randomly distributed into six groups: (1) Control (standard pellet diet), (2) ACE (standard pellet diet supplemented with 12% (*w*/*w*) of wild olive oil), (3) EVOO (standard pellet diet supplemented with 12% of extra virgin olive oil), (4) L-NAME (mice made hypertensive following treatment with 45 mg L-NAME/kg/day), (5) LN + ACE (L-NAME-treated hypertensive mice fed the same diet as in group 2; and (6) LN + EVOO group (L-NAME-treated hypertensive mice fed the same diet as in group 3). All treatments lasted 6 weeks under continuous monitoring of solid and liquid intake. Animals were housed under standard regulated conditions (23 ± 1 °C, 12 h/12 h light/dark cycles).

### 2.2. Dietary Supplementation

A commercial rodent chow (ROD14IRR, Sodispan Research, Altromin, Germany) was supplemented with 12% of ACE oil (groups 2 and 5) or EVOO (groups 3 and 6), as mentioned above. The specifications for the preparation of the diets and the chemical composition of ACE oil and EVOO were previously reported [19]. The oil-powder pellets were kept cool and protected from light until daily use.

Both oils were produced in Sierra de las Nieves (Málaga, Spain) using exactly the same extraction methods, in accordance with standard protocols to comply with extra virgin olive oil definition. Briefly, oils were obtained by a process of grinding, mixing and extraction at room temperature by centrifugation in a two-phase system. Then, they were kept in an insulated cellar with controlled temperature, protected from light and away from any source of external flavors. The oils were analyzed prior to their use in animal experiments [19]. The required amount of L-NAME in the water bottles was adjusted every week after monitoring each animal’s body weight and water intake; the specific dose was chosen from prior studies performed routinely in our laboratory.

### 2.3. Animal Characteristics

Systolic and diastolic blood pressure values (SBP, DBP) were recorded every week throughout treatment by the non-invasive, tail-cuff occlusion method in conscious animals by means of a pressure recorder (NIPREM 645, CIBERTEC S.A., Madrid, Spain). Blood pressure values were pooled and averaged from three to four consecutive measurements.

### 2.4. Electroretinography (ERG)

Dark-adapted mice (12 h) were anaesthetized, under dim red light, with an intraperitoneal injection of a mix of ketamine (Ketamidor, Richter Pharma AG, Wels, Austria; 100 mg/mL) and xylazine (Xilagesic, CALIER, Barcelona, Spain; 20 mg/mL) in saline solution (NaCl 0.9%), at a final concentration of 0.5 mL/150 g body weight. The animals’ temperature was kept at 37 °C with a water-content heating pad, to avoid electric noise during the recording. Pupil dilation was achieved with 1% tropicamide (Alcon Cusí S.A., El Masnou, Barcelona, Spain). A needle was located at the base of the tail for grounding and a reference electrode was placed on the tongue. A gold band electrode was used to record ERGs from the right eye. The electrode was placed on the cornea, and a drop of 2% methyl-cellulose (Methocel 2%, Omnivision, Neuhausen, Switzerland) was placed between the cornea and the electrode to assure electrical conductivity and to protect the eye.

Full-field flash ERG was performed with a Ganzfeld dome. Initially, a first scotopic phase was performed with flashes of increasing intensity (−4.0, −3.0, −2.0, −1.5, −1.0, −0.5, −0.0, 0.5, 1.0, and 1.5 log cd·s^−1^·m^−2^). The interval between flashes in scotopic conditions ranged from 1.2 s for dim flashes to 15 s for the highest intensity stimuli. ERG signals were amplified and filtered between 0.3 and 1000 Hz with a Grass amplifier (CP511 AC amplifier, Grass Instruments, Quincy, MA, USA, EE.UU.). In general, scotopic b-wave (b-scot) informs about rod-driven circuitry, mixed waves (mixed) indicate rod and cone photoreceptors (a-mix) and their postsynaptic circuitry (b-mix) activity, and photopic b-wave (b-photo) test how photopic conditions affect cone-driven circuitry through rod-saturating light stimulation. Moreover, oscillatory potentials (OP) were isolated using a bright flash (1.5 log cd·s^−1^·m^−2^) and band pass filter between 30 and 10,000 Hz. Cone-mediated responses were recorded on a rod-saturating background of 30 cd/m^2^, after 5 min of adaptation, with flashes of increasing intensity (−1.0, −0.5, −0.0, 0.5, 1.0 and 1.5 log cd·s^−1^·m^−2^). Under photopic conditions, the interval between light flashes was fixed at 1.2 s. Flicker (FL) response was recorded at different frequencies (20, 30 and 50 Hz) and an intensity of 1.5 log cd·s^−1^·m^−2^. ERG wave components were measured manually using the commercial software LabChart Pro v.8.1.13 (ADInstruments Ltd., Oxfordshire, UK).

### 2.5. Sample Harvesting

Animals were anesthetized with intraperitoneal injections of ketamine (75 mg/Kg) plus diazepam (10 mg mg/Kg), then subjected to cervical dislocation. The retinas were immediately isolated under a binocular stereo microscope, immersed in liquid nitrogen and maintained at −80 °C until use for gene/protein expression analyses. For immunodetection of proteins of interest in retinal/choroidal tissue, eyes were processed as described below (Section 2.8).

### 2.6. Western Blotting Analyses

Retinal homogenates were prepared in protease inhibitor-containing phosphate buffer saline (50 mM PBS, Sigma Aldrich-Roche, Madrid, Spain) using a Potter-Elvehjem tissue grinder. Homogenized samples were centrifuged for 10 min at 10,000× *g* and aliquots of the corresponding supernatants were set aside to estimate the protein concentration according to the method described by Bradford [43]. Western blotting analyses were performed in retinal homogenates containing 40–50 μg of proteins, as previously described [19]. Specific primary and secondary antibodies are listed in Table 1. Blot signals were quantified by optical densitometry (Cytiva Europe GmbH, Barcelona, Spain), and constitutive β-actin was used as the loading control in all blots.

### 2.7. Real-Time PCR

Total RNA from each retina sample was isolated with TRIzol^®^ (Thermo Fisher Scientific, Madrid, Spain); then, reverse transcription reactions were carried out as described elsewhere [44]. Specific primers are listed in Table 2. Gene products were amplified in a CFX96 real-time PCR system (Bio-Rad, Madrid, Spain), and relative mRNA expression was quantified by the standard 2^−ΔΔCt^ method, using glyceraldehyde-3-phosphate dehydrogenase (GAPDH) as the housekeeping gene.

### 2.8. Immunohistofluorescence

Paraffin-embedded sections (5 μm thick) were obtained following intravitreal administration of 4% paraformaldehyde (PFA) in PBS; eyes were then post-fixed in 4% PFA for 24 h. The localization of PPAR isoforms (PPARγ and PPARα) in the retina and choroid was evaluated by immunohistofluorescence staining after deparaffination of the sections. A heat retrieval solution (Diva Decloaker, Biocare Medical, LLC, Pacheco, CA, USA) was used prior to incubation with specific primary antibodies (Table 3). Goat anti-mouse Alexa Fluor^®^ 647 (Cat. No. CSA3808) or Goat anti-rabbit Alexa Fluor^®^ 488 (Cat. No. CSA3211), where appropriate, were chosen as fluorescent secondary antibodies, and DAPI Fluoromount-G^®^ was used as a nuclear/chromosomal counterstain.

### 2.9. Statistical Analyses

Results are expressed as means ± standard error of the mean (SEM). GraphPad InStat Software (v. 3.10, San Diego, CA, USA) was used to run one-way analysis of variance (ANOVA) followed by post-hoc Tukey’s multiple comparison test, and *p* < 0.05 was considered statistically different.

## 3. Results

### 3.1. Validation of the Experimental Approach

**Figure 1:**
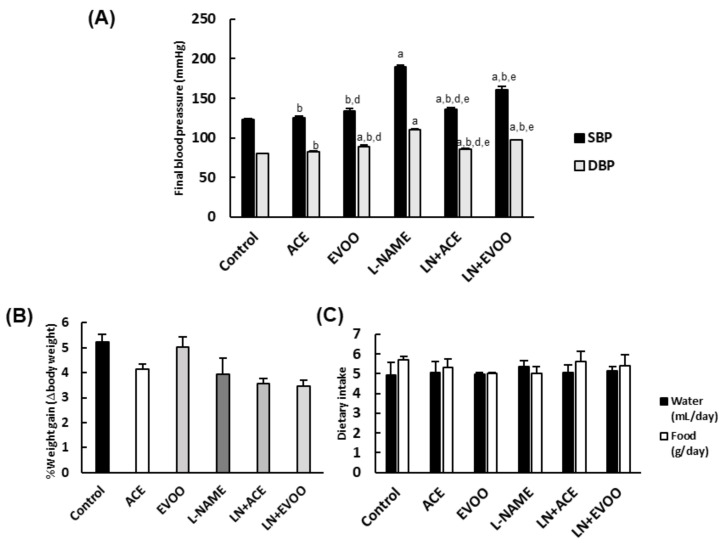
General parameters. (**A**) Final blood pressure, (**B**) weight gain and (**C**) liquid and solid diet intake in the six experimental animal groups. Values are expressed as mean ± SEM of seven animals per group: ^a^ *p* < 0.05 vs. Control; ^b^ *p* < 0.05 vs. L-NAME; ^d^ *p* < 0.05 vs. LN + EVOO; ^e^ *p* < 0.05 vs. ACE.

At the end of the 6-week experimental period, SBP/DBP values showed a significant (*p* < 0.05) rise of both parameters in the L-NAME group (189/110 mmHg, respectively), in comparison with all other animal groups (Figure 1A). Dietary supplementation with ACE oil counteracted the typical effect of L-NAME, such that the values recorded in the LN + ACE group fell slightly below the hypertensive threshold (136/85 mmHg). On the other hand, the blood pressure lowering effect of EVOO was milder than that of ACE oil (161/97 mmHg for LN + EVOO group). As shown in Figure 1A, the intake of either oil had no effect in normotensive (L-NAME-free) mice (126/83 and 134/89 mmHg for ACE and EVOO groups, respectively) when compared with the Control group (123/80 mmHg). In addition, no differences were observed among any of the study groups in terms of weight gain (Figure 1B) or solid/liquid intake (Figure 1C).

### 3.2. Retinal Function Analyzed by Full-Field ERG

**Figure 2:**
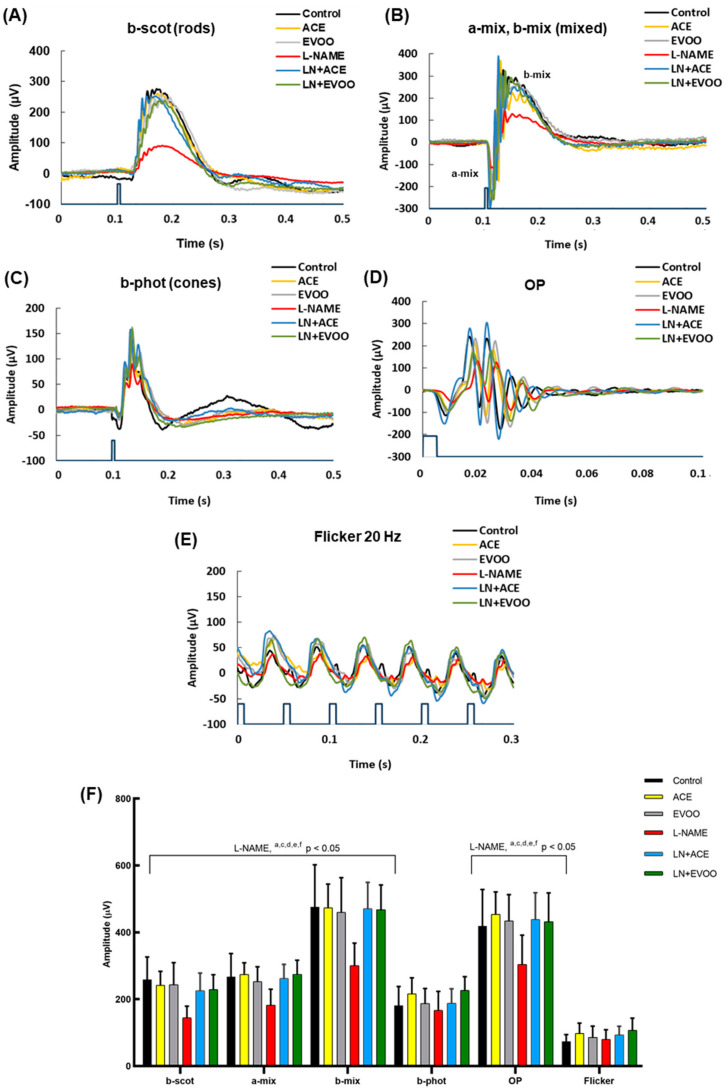
Electroretinogram (ERG) responses. Waveforms of representative ERG recordings: (**A**) b-scot (rods), (**B**) a-mix, b-mix (mixed), (**C**) b-phot (cones), (**D**) oscillatory potentials (OP) and (**E**) flickers of each experimental group are represented. (**F**) Mean amplitude of all waves from the different experimental groups. Values are expressed as mean ± SEM of ten animals per group: ^a^ *p* < 0.05 vs. Control; ^c^ *p* < 0.05 vs. EVOO; ^d^ *p* < 0.05 vs. LN + EVOO; ^e^ *p* < 0.05 vs. ACE; ^f^ *p* < 0.05 vs. LN + ACE.

Waveforms of the different recording conditions are depicted in Figure 2A–E. The analyses of these responses to light recorded after overnight dark-adaptation showed a significant (*p* < 0.05) reduction in amplitudes of scotopic waves b-scot (rods), a-mix and b-mix (mixed), and oscillatory potentials (OP), in the L-NAME group in comparison with Control animals (Figure 2F). Although no significant differences were observed in the b-phot amplitude, indicating no modifications in retinal activity of cones, and flickers (FL), a decreasing trend in its amplitude secondary to treatment with L-NAME could be extracted from our results. Regarding the groups fed with oil-enriched diets, no disturbances were found in LN + ACE and LN + EVOO animals, thus demonstrating the efficacy of both oil diets to prevent the negative effects of L-NAME on retinal function. In addition, no differences were found between any of the oil-supplemented groups (with or without the hypertensive phenotype), which means that no better efficacy is displayed between the ACE oil and EVOO diets in terms of visual retinal function.

### 3.3. PPARs Expression in Retina Layers

**Figure 3:**
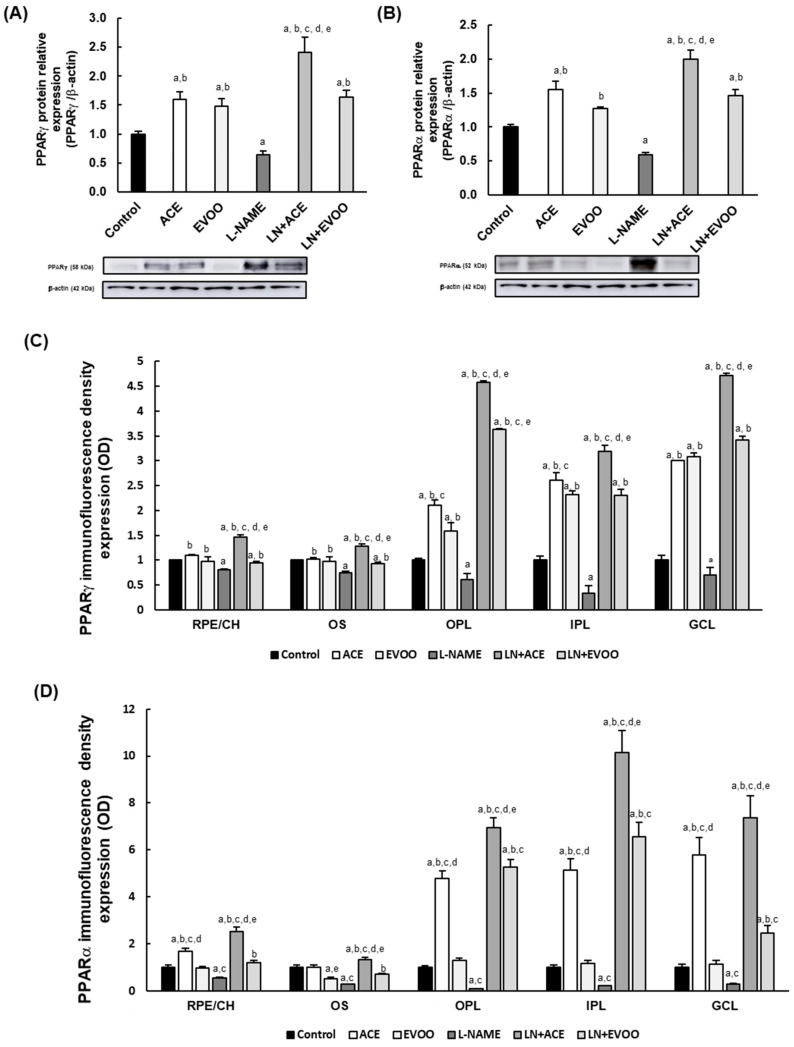

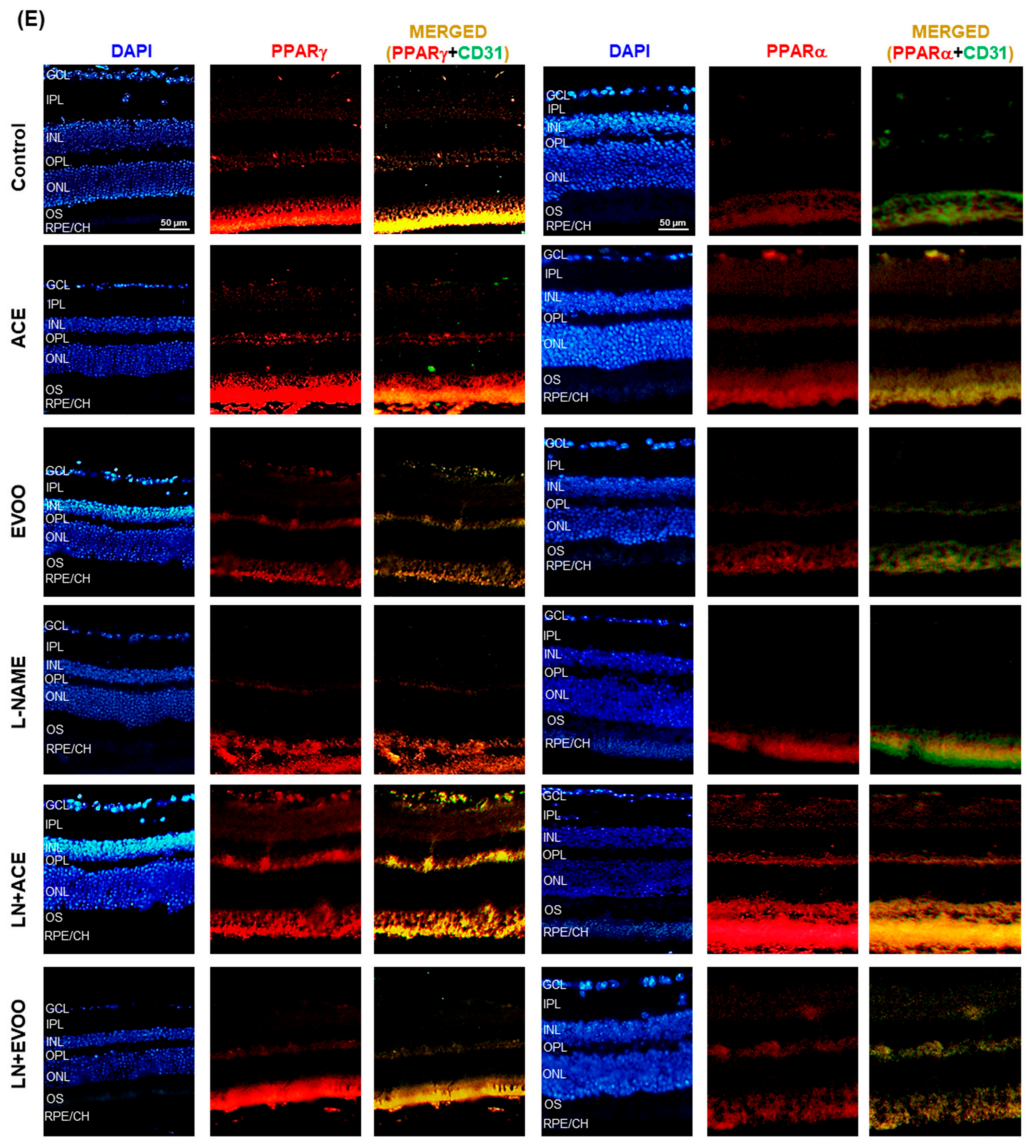
Protein expression of (**A**) PPARγ and (**B**) PPARα in retina homogenates. Quantitative analyses of fluorescence signal of (**C**) PPARγ and (**D**) PPARα in the retinal layers and choroid, and (**E**) representative fluorescence signal of PPARγ (left) and PPARα (right), as indicated. Panels show PPAR expression (red) and double staining with CD-31 (green) in the different retinal layers, with the merge visible in yellow color. Nuclei staining with DAPI (blue color) was used to identify the different retinal layers in each experimental group. Magnification: 10×. Values are expressed as mean ± SEM of five animals per group: ^a^ *p* < 0.05 vs. Control; ^b^ *p* < 0.05 vs. L-NAME; ^c^ *p* < 0.05 vs. EVOO; ^d^ *p* < 0.05 vs. LN + EVOO; ^e^ *p* < 0.05 vs. ACE. GCL: ganglion cell layer; IPL, inner plexiform layer; INL, inner nuclear layer; OPL, outer plexiform layer; ONL, outer nuclear layer; OS, outer segments; RPE/CH, retinal pigmentary epithelium/choroid.

Peroxisome proliferator-activated receptor (PPAR) γ and PPARα expression and localization were quantified by Western blotting and by immunofluorescence. The hypertensive L-NAME group showed a significant (*p* < 0.05) decrease of both PPARγ (36%) and PPARα (42%) expression compared to the Control group (Figure 3A,B). Although normotensive animal groups fed ACE oil displayed upregulation of PPAR isoforms (1.6-fold), the LN + ACE group showed even higher overexpression of these receptors (2.4- and 2-fold for PPARγ and PPARα, respectively). PPARγ and PPARα were also upregulated in EVOO (1.5- and 1.3-fold, respectively) and in LN + EVOO (1.6- and 1.5-fold, respectively) groups relative to normotensive animals. However, when comparing the global effects of ACE oil and EVOO, a significantly (*p* < 0.05) greater capacity to upregulate PPARs was observed in the former.

Quantification of immunofluorescence signal of PPARs in the retinal layers yielded similar results to those obtained from Western blotting analysis (Figure 3C,D). As shown in Figure 3E, PPARγ and PPARα expression was localized at the ganglion cell layer (GCL), inner plexiform layer (IPL), outer plexiform layer (OPL), outer segments (OS) and retinal pigment epithelium/choroid (RPE/CH). Fluorescence signals dropped drastically for both PPARγ (19%, 25%, 30%, 67% and 31% in RPE/CH, OS, OPL, IPL and GCL, respectively) and PPARα (47%, 72%, 91%, 79% and 72% in the same respective layers) in retinal sections obtained from L-NAME-treated animals, in comparison with the Control group. Oil-supplemented groups displayed a clearly visible increase in the retinal expression of PPARs, with a more prominent effect in the case of ACE oil-fed hypertensive animals (PPARγ: 1.5-, 1.3-, 4.6-, 3.2- and 4.7-fold increase; PPARα: 2.5-, 1.3- 6.9-, 6.5- and 7.4-fold increase in RPE/CH, OS, OPL, IPL and GCL, respectively, in comparison to Control group). In turn, upregulation of PPARs (relative to Control group) was only noted in some retinal layers in hypertensive mice fed EVOO (e.g., PPARγ expression increased 3.6-, 2.3- and 3.4-fold, and PPARα increased 5.3-, 6.5- and 2.44-fold, in OPL, IPL and GCL, respectively). Interestingly, PPARα immunofluorescence signal was generally higher than that of PPARγ in the different animal groups. In addition, PPARγ seems to yield higher signals in GCL and OPL, whereas PPARα signal was more abundant in IPL and OPL. Since the endothelial marker (anti-CD31) co-localized with PPAR immunofluorescence signals, these experiments confirmed the relevance of endothelial retinal cells in PPAR release.

### 3.4. Inflammatory Biomarkers in the Retina

**Figure 4:**
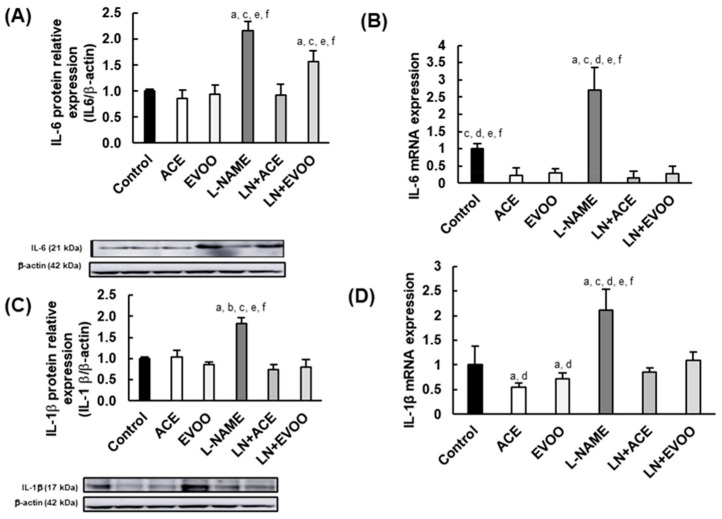

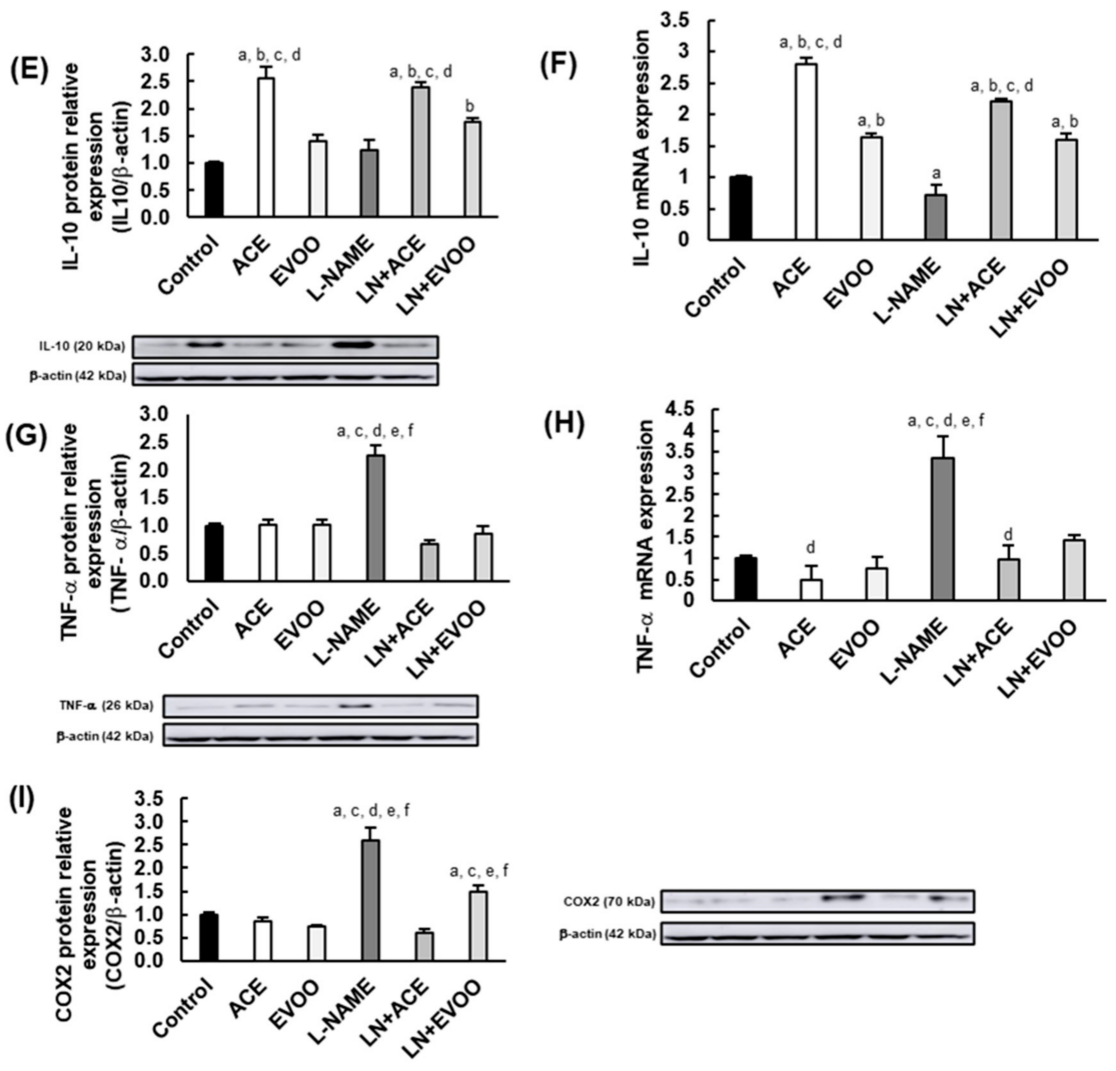
Protein and gene expression of inflammation-related biomarkers IL-6 (**A**,**B**), IL-1β (**C**,**D**), IL-10 (**E**,**F**), TNF-α (**G**,**H**) and COX2 (**I**) in retina homogenates. Gene expression was quantified as relative to GAPDH in each corresponding group. Values are expressed as mean ± SEM of five animals per group: ^a^ *p* < 0.05 vs. Control; ^b^ *p* < 0.05 vs. L-NAME; ^c^ *p* < 0.05 vs. EVOO; ^d^ *p* < 0.05 vs. LN + EVOO; ^e^ *p* < 0.05 vs. ACE; ^f^ *p* < 0.05 vs. LN + ACE.

In retinal homogenates, L-NAME-treated mice presented with upregulation of protein/gene expression of IL-6 (2.2- and 2.7-fold for protein and mRNA levels, respectively), IL-1β (1.8- and 2.1-fold, respectively), TNF-α (2.3- and 3.3-fold, respectively), and COX2 (2.6-fold), when compared to the Control group (Figure 4A–D,G–I). Despite no significant changes seen in IL-10 protein expression between Control and L-NAME groups, the gene expression was significantly lower (29% reduction; *p* < 0.05) in the latter (Figure 4E,F).

Focusing on olive oil-enriched diets, ACE oil and EVOO-fed hypertensive animals benefited from reduced IL-6 gene and protein expression up to values similar or even lower than those found in control normotensive animals, which supports the competence of both oils to reduce the levels of this cytokine locally in the retina. Similar results were obtained regarding the expression of IL-1β (Figure 4C,D), and no differences were found between LN + ACE and LN + EVOO groups with respect to these two pro-inflammatory cytokines, except for IL-6 protein expression that was significantly lower in the former (Figure 4A). In contrast, ACE oil induced substantial upregulation of anti-inflammatory IL-10 in both L-NAME-free (2.5- and 2.7-fold for protein/mRNA expression, respectively) and L-NAME co-administered mice (2.9-/2.2-fold), an effect that was not observed either in EVOO or in LN + EVOO groups (Figure 4E,F).

The similar expression of TNF-α found in Control, ACE, EVOO, LN + ACE and LN + EVOO groups was much lower than the values of the L-NAME group (Figure 4G,H). Concerning COX2, the two oil diets downregulated its expression in hypertensive mice, but the reduction was greater in LN + ACE than in LN + EVOO group (200% vs. 111% relative to L-NAME, respectively). No significant changes were found in this regard between oil-free (Control) and oil-fed (ACE, EVOO) normotensive animals.

## 4. Discussion

The model of L-NAME-treated rodents has been widely used for in vivo induced experimental arterial hypertension (AH). SBP and DBP values obtained at the end of the treatment indicate the presence of severe hypertension in our study design, according to current guidelines on AH [45]. As previously reported, no significant (*p* > 0.05) effects on feeding behavior or weight gain were observed between normotensive and hypertensive mice after a 6-week period of feeding oil-enriched diets [19]. Our research group was pioneer in describing a blood pressure lowering effect of ACE oil in mice treated with L-NAME, showing a preferential action of this “wild origin” oil compared to a parallel EVOO-enriched diet. Our former analyses showed that ACE oil presented higher content in sterol, tocopherols, triterpene acids, alcohols, and secoiridoids than an EVOO of similar origin/characteristics. Therefore, the reduction of SBP and DBP could be partly mediated by these minor compounds with known antioxidant properties [46,47,48,49]. Specifically, the high content of secoiridoid compounds observed in ACE oil led us to think that these compounds might be responsible for the greater hypotensive effect observed in hypertensive animals fed this particular oil.

The importance of AH as a risk factor for many pathologies, including neurological disorders [50] and regrettably widespread hypertensive retinopathy, warrants exploring how retinal function is affected in L-NAME-induced arterial hypertension. In that experimental group, our ERG recordings exhibited a remarkable reduction in amplitudes of b-scot (rod functionality), b-mix and a-mix waves, and a sightly reduction in OP, but no differences were attributable to AH in b-phot (cone activity) and FL waves. These findings are in agreement with previous assessment of visual function in spontaneously hypertensive rats (SHR), where b-scot amplitude was also reduced [51]. Our diminished OP amplitudes are also in line with Negretto et al. [52], who reported smaller OP in hypertensive patients, thus suggesting that the inner retina might be affected by AH. Previous morphometric analysis in the retina of L-NAME-treated animals showed thinner GCL, OS and RPE/CH layers [19,53], which might be associated with arterial sclerosis and vascular contraction in choroid [54], as well as with the reduction of retinal blood flow described in hypertensive patients [55]. Since b-scot wave and OP responses inform about inner retinal cell layers, mainly the activity of amacrine and bipolar cells, while a-wave reflects the activity of photoreceptors, specific modifications of the ERG-pattern in L-NAME-administered mice could be explained by the presence of retinal vascular dysfunction due to AH, which, in turn, would affect both the inner retina and OS layer. Moreover, since modifications of a-wave amplitude have been related to loss of photoreceptors [56], the reduction in the thickness of the OS and RPE/CH layers found in L-NAME-induced hypertensive animals suggests the existence of damaged photoreceptors with compromised retinal function in our animal model of AH. Interestingly, both ACE oil and EVOO diets were able to reverse the ERG alterations seen in the L-NAME group, reaching patterns like those found in the Control group. This supports the notion that diets containing ACE oil- and EVOO could be postulated as nutraceutical tools to counteract AH-related retinal dysfunction.

As mentioned above, there is a great deal of evidence concerning the healthy properties of olive oil components based on the modulation of oxidative stress and inflammation; unfortunately, the literature about specific effects of EVOO on hypertensive retinas is very limited, and studies on the general properties and healthy potential of ACE oil are even scarcer. After evaluating the antioxidant properties of ACE oil in hypertensive retinas [19], we decided to analyze the possible anti-inflammatory properties derived from the daily consumption of this oily product. In this sense, we first focused on the role of nuclear receptors PPARs. PPARs are involved in the control of metabolic activities through differential regulation of glucose and lipid metabolism following activation by fatty acids [57], which makes them attractive targets for oil-mediated regulation. Moreover, PPARs can reduce excess inflammation by transcriptional regulation of a wide variety of genes, not only those related to inflammation but also those involved in metabolism, proliferation and differentiation pathways [58], and they can even regulate neurotoxic activities related to microglia activation [58]. PPAR expression and activation provides clear beneficial effects on different retinopathies of vascular origin [58]. These pleiotropic effects have prompted the use of different PPAR agonists to treat various inflammatory retinopathies, including DR, oxygen-induced retinopathy and AMD [59,60,61]. In this regard, PPARs have been shown to downregulate the expression of pro-inflammatory biomarkers in retinopathy microvascular dysfunction, as well as to regulate endothelial cell function by targeting angiogenesis [62,63].

In the current study, the clear reduction of PPARγ and PPARα expression found in the L-NAME group indicates that the retinal inflammatory profile could be affected in arterial hypertension via PPAR inhibition, an alteration that could be prevented with ACE oil and with EVOO-enriched diets (thus supporting a beneficial anti-inflammatory strategy via PPAR upregulation), with better performance in the case of ACE oil. In addition, the colocalized expression of both PPAR isoforms with CD-31 in GCL, IPL, OPL, OS retinal layer and in RPE/CH confirms the relationship between PPARs and endothelial cells. Preliminary studies carried out in our laboratory also confer beneficial effects to ACE oil in terms of improving endothelial dysfunction in aortas from L-NAME treated rats (unpublished data); interestingly, vascular reactivity in the aorta recovered in parallel with a reduction of endothelial inflammatory biomarkers, supporting the idea that ACE oil could also modulate retinal/choroidal endothelial cell function. Both olive oil and some of its minor components, such as polyphenols, have been described as useful regulators of PPAR expression [64,65], although regulation of PPAR expression in the hypertensive retina has not previously been reported in animal studies or in humans.

In view of this interesting regulation on PPAR isoforms, and to further characterize the anti-inflammatory capacity of olive oils in the retina, we postulated that the profile of major inflammation-related biomarkers would be unbalanced in our experimental AH setting. Indeed, pro-inflammatory biomarkers (IL-6, IL-1β, TNF-α and COX2) were all abnormally elevated in hypertensive animals, a situation that was reversed after the simultaneous administration of oil-enriched diets. On the other hand, the protein and gene expression of anti-inflammatory IL-10 was highly upregulated by the oil diets, especially after ACE oil consumption. Interventions to reduce inflammation have been used in many retinopathies as a way to prevent and/or to treat retinal damage. Thus, some components of EVOO, such as omega-3 fatty acids, have been explored against retinopathy of prematurity (ROP) [66], as have polyphenols and flavonoids in DR [67]. In the current study, the slight differences found between ACE oil and EVOO in this regard might be ascribed to a different proportion of minor components. As mentioned above, tocopherols, triterpene acids and polyphenols, mainly secoiridoids, are found in higher proportion in ACE oil, and these components have been attributed important anti-inflammatory properties. For instance, oleocanthal, one of the major secoiridoids found in ACE oil, has antioxidant and anti-inflammatory properties that could help reduce glial activation and inflammatory biomarkers [68,69]. Triterpene acids might also account for a preferential anti-inflammatory effect in ACE oil compared to EVOO. Oleanolic acid seem to have neuroprotective effects through modulation of inflammatory biomarkers (e.g., IL-6, IL-1β, TNF-α) and nitric oxide (NO) bioavailability [70,71]; and, together with ursolic acid, they were postulated as a promising anti-inflammatory compounds [42]. In the same way, maslinic acid reduced the inflammatory response through regulation of IL-1β [72] and COX-2 reduction, among other inflammatory biomarkers [73]. Moreover, not only did these compounds reduce pro-inflammatory biomarkers, but they also increased IL-10 levels [74,75,76].

In summary, ACE oil and EVOO exerted a retinoprotective effect in hypertensive mice based upon their anti-inflammatory properties, which adds value to their antioxidant effect recently demonstrated [19]. Our study offers a broad vision of how these extra-virgin olive oils can regulate inflammatory events in L-NAME-treated animals, counteracting the retinal dysfunction observed in ERG recordings in a hypertensive context. Although ACE oil tends to show better results than EVOO due to a higher content of some minor components, further experiments with specific molecules contained in olive oil are necessary to clarify these healthy effects on hypertensive retinas.

## 5. Conclusions

The present work describes changes on retinal function and inflammatory events in the retina and choroid of a mouse model of L-NAME-induced hypertension. A greater anti-inflammatory effect seems to be attributable to ACE (wild olive) oil in hypertension-related ocular diseases in comparison with a reference EVOO of similar geographic origin and extraction method. A different profile between ACE oil and EVOO regarding minor components might explain the better outcomes in favor of the former. We postulate ACE oil-enriched diets as a novel nutraceutical tool to abolish AH-related retinal anti-inflammatory damage. Considering also its demonstrated antioxidant capacity, ACE oil could represent a promising therapeutical approach to prevent and/or to treat retinal pathologies, including hypertensive retinopathy, AMD, diabetic retinopathy and other vascular retinopathies.

## 6. Patents

The retinoprotective effect of acebuche oil was previously applied for patent by the authors (application number P202030625) on 23 June 2020, at the Oficina Española de Patentes y Marcas (OEPM), Ministerio de Industria, Comercio y Turismo.

## Figures and Tables

**Table 1:** Antibodies used for Western blotting analysis. SCB = Santa Cruz Biotechnology (Santa Cruz, CA, USA).

Primary Antibody	Origin	Dilution	Secondary Antibody	Dilution	Reference
Anti-PPARγ	Mouse monoclonal	1:2000	Goat Anti-Mouse	1:4000	SCB
Anti-PPARα	Mouse monoclonal	1:2000	Goat Anti-Mouse	1:4000	SCB
Anti-IL-6	Mouse monoclonal	1:1000	Goat Anti-Rabbit	1:2000	SCB
Anti-IL-1β	Mouse monoclonal	1:1000	Goat Anti-Mouse	1:2000	SCB
Anti-IL-10	Mouse monoclonal	1:1000	Goat Anti-Mouse	1:2000	SCB
Anti-TNF-α	Mouse monoclonal	1:1000	Goat Anti-Mouse	1:2000	SCB
Anti-COX2	Mouse monoclonal	1:1000	Goat Anti-Mouse	1:2000	SCB
Anti-β-Actin	Mouse monoclonal	1:20,000	Goat Anti-Mouse	1:30,000	SCB

**Table 2:** Primers used for real-time PCR.

Gene	Forward Primer (5′→ 3′)	Reverse Primer (5′→3′)
IL-6	CTCTGCAAGAGACTTCCATCC	TTCTGCAAGTGCATCATCGT
IL-1β	CCGTGGACCTTCCAGGATGA	GGGAAGGTCACACACCAGCA
IL-10	CTGGACAACATACTGCTAACCG	GGGCATCACTTCTACCAGGTAA
TNF-α	CCACGCTCTTCTGTCTACTG	ACTTGGTGGTTTGCTACGAC
GAPDH	GCCAAAAGGGTCATCATCTCCGC	GGATGACCTTGCCCACAGCCTTG

**Table 3:** Primary antibodies used for immunofluorescence studies.

Primary Antibody	Origin	Dilution	Reference
Anti-PPARγ	Mouse monoclonal	1:200	Santa Cruz Biotechnology, Santa Cruz, CA, USA
Anti-PPARα	Mouse monoclonal	1:200	Santa Cruz Biotechnology
Anti-CD31	Rabbit monoclonal	1:200	Rockland Immunochemicals, Limerick, PA, USA

## Data Availability

The data presented in this study are available in the article.
